# Patterns of Online Medical Crowdfunding in India

**DOI:** 10.1001/jamanetworkopen.2024.54855

**Published:** 2025-01-16

**Authors:** Manraj Singh Sra

**Affiliations:** 1 Division of Hematology, Mayo Clinic, Rochester, Minnesota

## Abstract

This cross-sectional study of online fundraising campaigns for medical expenses conducted in India examines the distribution of fundraisers by amount raised and disease type.

## Introduction

Online medical crowdfunding in India is growing by 25% annually, with medical fundraising campaigns receiving nearly $630 million in contributions.^1^ However, the diseases for which patients seek crowdfunding in India and the financial support provided remain unexplored.

The primary objective of this study was to examine disease patterns in online medical crowdfunding in India. Secondary objectives included analyzing campaign differences across disease categories, geographical distribution of campaigns, and the association between state-level household medical expenditure (HME) and campaign outcomes.

## Methods

Data for this cross-sectional study were obtained from Ketto, an online medical crowdfunding platform that hosts nearly 20% of campaigns in India.^1,2^ Medical campaigns were identified as campaigns listed under the Medical category, with additional campaigns found through keyword searches in other categories. The analysis included campaigns launched from February 1, 2020, to December 31, 2022. For each campaign, the following data were extracted: campaign text, start and end dates, recipient’s name, location, target amount, amount raised, and campaign-related marketing features. The detailed methodology for data collection and extraction has been described elsewhere.^3^ This study was exempt from ethical clearance by the ethics committee of the Indian Institute of Management Bangalore and adhered to the Strengthening the Reporting of Observational Studies in Epidemiology (STROBE) reporting guideline. Informed consent was not required as the data were publicly accessible, and there was no interaction or intervention with the individuals posting the campaigns.

Similar to previous studies, campaigns were classified into disease categories: acute illness, cancer, cardiovascular, coronavirus disease (COVID-19), kidney, neurological, transplant, trauma, or others^4^ (eTable in Supplement 1). Categorical data were summarized as counts and percentages, while continuous variables were presented as mean (SD) and median (IQR) values. Contributions are reported in US dollars. State-level mean HME was based on the National Sample Survey Organization’s Social Consumption: Health survey (75th round).^5^ The correlation between state-level crowdfunding metrics and HME was assessed using Spearman correlation. Sensitivity analysis included only states with more than 50 campaigns. Two-tailed *P* < .05 was considered statistically significant. All analysis was performed in R version 4.1.2 (R Project for Statistical Computing) from August 2024 to September 2024.

## Results

A total of 2440 medical crowdfunding campaigns were included. Cancer (620 campaigns [25.4%]) was the most common cause throughout the study period and in the majority of the states (Table; Figure, panel A). The highest total annual amounts raised ($6 623 936) and requested ($25 068 068) were in 2021 (Figure, panel B). The highest median (IQR) amount requested and raised was for campaigns in the other category ($19 648 [$9526-$29 769]) and COVID-19 ($3193 [$1783-$6731]), respectively. COVID-19 campaigns received the highest percentage of requested funds (median [IQR], 45% [23%-81%]), with 18.6% of campaigns meeting their goals.

**Table.** Characteristics of Medical Crowdfunding Campaigns Stratified by Disease Categories

Characteristic	Overall (n = 2440)	Acute illness (n = 291)	COVID-19 (n = 279)	Cancer (n = 620)	Cardiovascular (n = 116)	Neurological (n = 337)	Other (n = 331)	Kidney (n = 259)	Transplant (n = 38)	Trauma (n = 169)
Year of publishing campaign, No. (%)										
2020	486 (19.9)	32 (11.0)	38 (13.6)	136 (21.9)	29 (25.0)	61 (18.1)	81 (24.5)	58 (22.3)	15 (39.5)	36 (21.3)
2021	1211 (49.6)	172 (59.1)	238 (85.3)	272 (43.9)	44 (37.9)	153 (45.4)	147 (44.4)	115 (44.4)	11 (29)	59 (34.9)
2022	743 (30.5)	87 (29.9)	3 (1.1)	212 (34.2)	43 (37.1)	123 (36.5)	103 (31.1)	86 (33.2)	12 (31.6)	74 (43.8)
Duration of campaign, d										
Mean (SD)	80 (116)	51 (46)	42 (62)	101 (125)	73 (136)	86 (141)	79 (120)	100 (125)	101 (154)	76 (114)
Median (IQR)	45 (45-75)	45 (39-46)	45 (15-45)	48 (45-102)	45 (27-47)	45 (45-70)	45 (40-67)	46 (45-95)	45 (45-94)	45 (42-51)
Gender, No, (%)										
Female	826 (33.9)	92 (31.6)	91 (32.6)	260 (41.9)	36 (31.0)	108 (32.1)	113 (34.1)	78 (30.1)	9 (23.7)	39 (23.1)
Male	1614 (66.2)	199 (68.4)	188 (67.4)	360 (58.1)	80 (69.0)	229 (68.0)	218 (65.9)	181 (69.9)	29 (76.3)	130 (76.9)
Amount requested, $										
Mean (SD)	18 944 (30 183)	19 357 (23 486)	14 416 (17 686)	20 153 (31 664)	12 738 (15 170)	20 222 (49 932)	24 839 (26 674)	15 895 (12 904)	28 013 (34 569)	14 072 (28 408)
Median (IQR)	11 908 (5954-23 815)	11 908 (6889-23 815)	9526 (5954-17 861)	11 908 (5954-23 815)	7145 (4168-16 522)	11 908 (5954-17 861)	19 648 (9526-29 769)	11 908 (7145-23 220)	16 075 (11 908-29 769)	9526 (5954-14 884)
Amount raised, $										
Mean (SD)	4859 (9410)	4744 (6048)	6048 (7583)	5130 (11 463)	3722 (5089)	4289 (7455)	5663 (11 054)	3624 (4524)	8753 (31 057)	3457 (4130)
Median (IQR)	2513 (1536-4905)	2823 (1715-5282)	3193 (1783-6731)	2321 (1510-4680)	2147 (1462-4032)	2580 (1562-4567)	2584 (1462-5294)	2521 (1537-4131)	2058 (1659-4051)	2107 (1351-4021)
Proportion of requested amount received, %										
Mean (SD)	36 (33)	36 (34)	54 (36)	32 (31)	41 (37)	36 (35)	30 (32)	30 (28)	22 (27)	35 (30)
Median (IQR)	25 (12-51)	25 (13-50)	45 (23-81)	23 (10-46)	29 (12-56)	26 (12-48)	18 (7-46)	21 (11-38)	14 (7-25)	25 (13-49)
Campaigns receiving target amount, No. (%)	180 (7.4)	21 (7.2)	52 (18.6)	34 (5.5)	16 (13.8)	22 (6.5)	13 (3.9)	9 (3.5)	3 (7.9)	10 (5.9)

**Figure.**
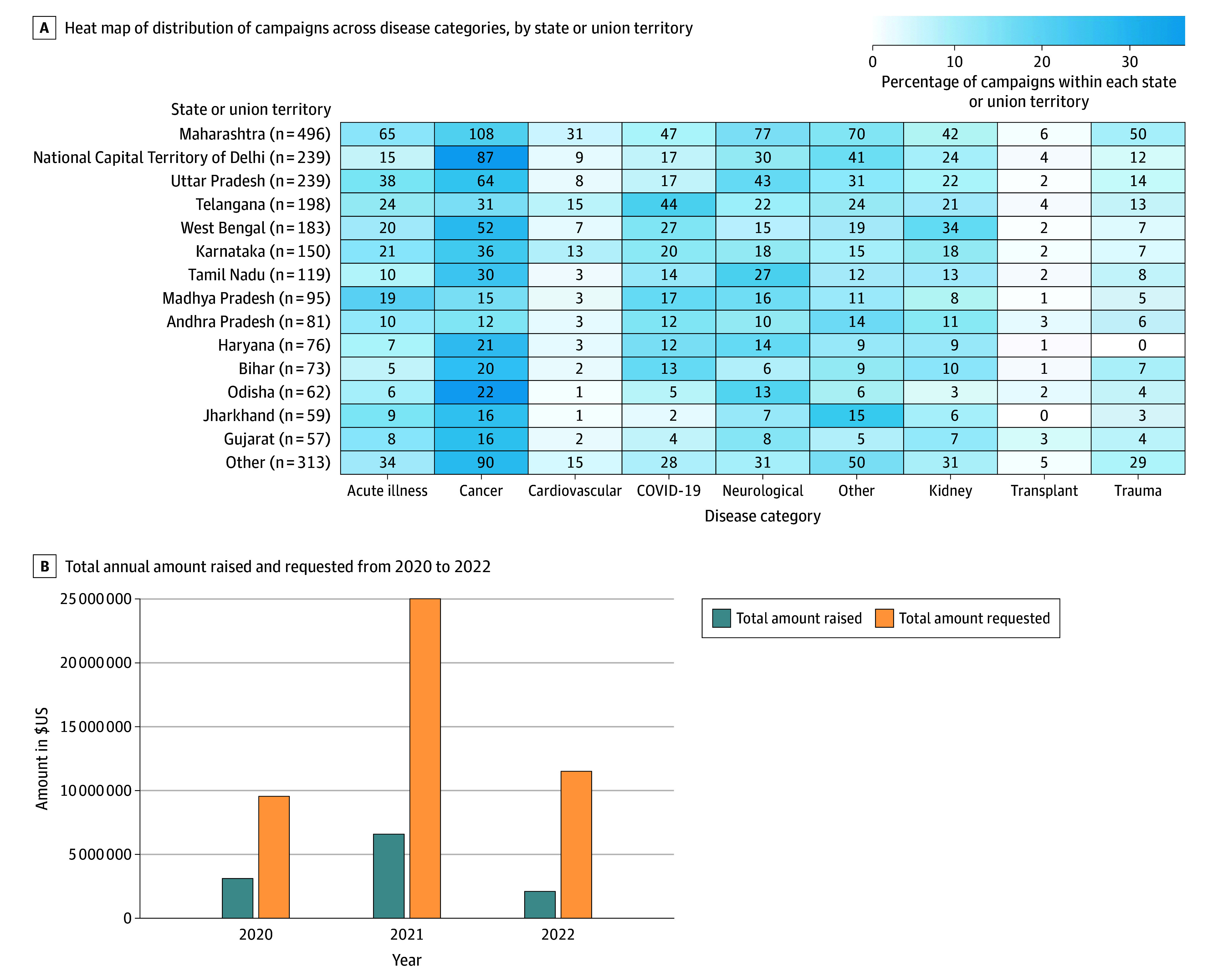
Heat Map of Campaigns by Disease Category and Totals Raised by Campaigns A, Regions with more than 50 campaigns are shown, with states or union territories with less than 50 campaigns grouped under other. The number in each box indicates the number of campaigns.

State-level mean HME correlated with the number of campaigns per 100 000 (*r* = −0.35; *P* = .04) and the mean amount raised (*r* = 0.4; *P* = .02), but not with the mean amount requested (*r* = 0.078; *P* = .66) or the percentage of requested funds received (*r* = 0.33; *P* = .053). In states with over 50 campaigns, only the correlation between HME and the amount raised remained significant (*r* = 0.6; *P* = .03).

## Discussion

Compared with high-income countries, India had lower rates of medical crowdfunding, with lower amounts requested and raised.^3^ This may be due to limited public awareness or support for crowdfunding. Cancer patients in India incur an average out-of-pocket expenditure of $4171, yet the median amount requested was $11 908, with a median amount raised of only $2321.^6^ This suggests that only patients with significant needs resort to crowdfunding, with limited success. Importantly, 7.4% of campaigns in India reached their target, compared with 33.4% in high-income countries.^3^ The higher success of COVID-19 campaigns may be due to public awareness and urgency.

Higher HME correlated with higher funds raised but fewer campaigns, possibly due to variations in awareness of crowdfunding resources. Limitations include data from a single platform and for only 3 years during the COVID-19 pandemic. Considering the high out-of-pocket expenditures and limited insurance coverage in India future studies should explore strategies to improve health care affordability.

## Notes

Supplement 1. **eTable.** Disease Category Definition

Supplement 2.
Data Sharing Statement
