# Supplementary material for: Patterns of Online Medical Crowdfunding in India
Source: JAMA Netw Open. 2025 Jan 16;8(1):e2454855. doi: 10.1001/jamanetworkopen.2024.54855 (PMC11739994; doi:10.1001/jamanetworkopen.2024.54855)
Supplement: Supplement 1. — eTable. Disease Category Definition [file jamanetwopen-e2454855-s001.pdf]

Supplemental Online Content

Sra MS. Pattern of online medical crowdfunding in India. *JAMA Netw Open.* 2025;8(1):e2454855. doi:10.1001/jamanetworkopen.2024.54855

eTable. Disease Category Definition

This supplemental material has been provided by the authors to give readers additional information about their work.

eTable. Disease category definition

Disease Category	Definition
Acute Illness	Campaigns associated with conditions with sudden onset, including but not limited to infections (except COVID-19), procedural complications, or pregnancy-related issues.
Cancer	Campaigns with keyword of cancer or linked to specific diagnosis of malignancy
Cardiovascular	Campaigns associated with heart or vascular related conditions
Coronavirus Disease (COVID-19)	Campaigns with keyword of COVID-19 or coronavirus disease.
Neurological	Campaigns associated with conditions related to brain, spinal-cord or peripheral nerves
Kidney	Campaigns associated with kidney related conditions
Transplant	Campaigns associated with organ transplantation or it's complication
Trauma	Campaigns associated with medical care for physical injuries
Other	Campaigns not covered among any of the above categories
