# Supplementary material for: Patterns of Online Medical Crowdfunding in India
Source: JAMA Netw Open. 2025 Jan 16;8(1):e2454855. doi: 10.1001/jamanetworkopen.2024.54855 (PMC11739994; doi:10.1001/jamanetworkopen.2024.54855)
Supplement: Supplement 2. — Data Sharing Statement [file jamanetwopen-e2454855-s002.pdf]

Data Sharing Statement

Data
Data available: Yes
Data types: Deidentified participant data
How to access data: Data for this study can be requested from the corresponding author (sra.manraj@mayo.edu).
When available: With publication

Supporting Documents
Document types: None

Additional Information
Who can access the data: Anyone requesting data
Types of analyses: Data will be shared for research proposals not involving direct human interaction or intervention with campaign organizers. No campaign organizer should be individually identified in any research outputs.
Mechanisms of data availability: Data will be made available with a signed data access agreement.
